# Heterosis May Result in Selection Favouring the Products of Long-Distance Pollen Dispersal in *Eucalyptus*

**DOI:** 10.1371/journal.pone.0093811

**Published:** 2014-04-21

**Authors:** João Costa e Silva, Brad M. Potts, Gustavo A. Lopez

**Affiliations:** 1 Centro de Estudos Florestais, Instituto Superior de Agronomia, Universidade de Lisboa, Lisboa, Portugal; 2 School of Biological Sciences and National Centre for Future Forest Industries, University of Tasmania, Hobart, Tasmania, Australia; The Australian National University, Australia

## Abstract

Using native trees from near the northern and southern extremities of the relatively continuous eastern distribution of *Eucalyptus globulus* in Tasmania, we compared the progenies derived from natural open-pollination (OP) with those generated from within-region and long-distance outcrossing. Controlled outcrossing amongst eight parents - with four parents from each of the northern and southern regions - was undertaken using a diallel mating scheme. The progeny were planted in two field trials located within the species native range in southern Tasmania, and their survival and diameter growth were monitored over a 13-year-period. The survival and growth performances of all controlled cross types exceeded those of the OP progenies, consistent with inbreeding depression due to a combination of selfing and bi-parental inbreeding. The poorer survival of the northern regional (♀_N_♂_N_) outcrosses compared with the local southern regional outcrosses (♀_S_♂_S_) indicated differential selection against the former. Despite this mal-adaptation of the non-local ♀_N_♂_N_ crosses at both southern sites, the survival of the inter-regional hybrids (♀_N_♂_S_ and ♀_S_♂_N_) was never significantly different from that of the local ♀_S_♂_S_ crosses. Significant site-dependent heterosis was detected for the growth of the surviving long-distance hybrids. This was expressed as mid-parent heterosis, particularly at the more northern planting site. Heterosis increased with age, while the difference between the regional ♀_N_♂_N_ and ♀_S_♂_S_ crosses remained insignificant at any age at either site. Nevertheless, the results for growth suggest that the fitness of individuals derived from long-distance crossing may be better at the more northern of the planting sites. Our results demonstrate the potential for early-age assessments of pollen dispersal to underestimate realised gene flow, with local inbreeding under natural open-pollination resulting in selection favouring the products of longer-distance pollinations. Indeed, heterosis derived from long-distance pollinations may be sufficient to counter local mal-adaptation, at least in the first generation.

## Introduction

Intra-specific gene flow links populations of a species, and provides a key source of genetic diversity to supplement and redistribute standing genetic variation within populations [1], [2]. Such redistribution is particularly important for many forest tree species where populations often exhibit strong-local adaptation, yet are facing rapid shifts in their climate niches over the next century [3], [4], [5], [6]. Seed and pollen both contribute to gene flow in forest trees, although most molecular-based estimates of their relative importance suggest that pollen is the dominant component of intra-specific gene flow, being most likely the common source of rare, long-distance dispersal events [5], [7], [8]. This is particularly the case in many temperate genera such as the wind-pollinated *Pinus* [9], [10] (but see [11]), *Quercus* [12] and *Populus* [13], as well as the animal-pollinated genera such as *Eucalyptus* [14], [15], [16]. However, while rare long-distance pollen dispersal has been reported from early life-history stages, and may be an important source of novel genes, dispersal may not correspond to realised gene flow [5], [17], [18]. For such pollen-mediated immigration to contribute to the standing genetic variation and evolutionary trajectory of the populations within a species, inter-population F_1_ hybrids must first survive to reproduction in competition with local genotypes. Thus, the fitness of inter-population F_1_ hybrids within a species is a key issue for understanding the potential for realised gene flow amongst differentially (co)adapted natural populations.

An optimum outcrossing distance is often observed in plants, due to a balance between outbreeding and inbreeding depression [19], [20], [21]. In inter-population or inter-specific crosses, outbreeding depression may be a consequence of mal-adaptation to ecological factors at the site of immigration (a dilution of adaptive parental genes due to hybridization), chromosomal rearrangements and/or incompatible epistatic interactions [22], [23], [24]. In contrast, limited seed and/or pollen dispersal may result in the build up of relatedness within local populations of a species, even with outbred or mixed mating systems [5], [23]. This is certainly the case in many forest tree species, where limited seed dispersal often results in forests comprising spatial mosaics of related individuals [25] which, when coupled with near-neighbour pollinations, can lead to significant bi-parental inbreeding at mating. Nevertheless, while there are exceptions, most forest tree species are predominantly outcrossing and appear to maintain high genetic loads of rare deleterious genes, resulting in reduced fitness of offspring arising from self-pollination or crosses with related individuals [26]. Such inbreeding depression may counter the development of local adaptation due to selection acting against the products of proximal matings, and thus may result in the pollen dispersal kernel underestimating realised gene flow [5].

The present study examines the potential for discrepancy between pollen dispersal and realised pollen-mediated gene flow in *Eucalyptus globulus* by evaluating the success of crosses between populations of *Eucalyptus globulus* subspecies *globulus* (hereafter referred to as *E. globulus*) growing near the extremes of its relatively continuous genetic and geographic distribution in south-eastern Tasmania [27], [28]. This globally-grown eucalypt species is native to south-eastern Australia, including the mainland and Tasmania, and has been one of the most extensively genetically studied of all eucalypt species. Most seed of *E. globulus* appears to be deposited within two canopy heights [29], [30], with molecular markers [31], [32] and crossing studies [33] clearly showing that the native forests comprise patches of related individuals. *E. globulus* has a mixed mating system and open-pollinated (OP) progenies exhibit significant inbreeding depression [34], [35], due to a combination of selfing (outcrossing rate ranges from 0.65 to 0.89 at the population level) and biparental inbreeding (up to 0.11) [36]. As with most eucalypts, gene flow in *E. globulus* is likely to be mainly pollen-mediated [16]. Although most pollen is still distributed in close proximity to a source, the pollen dispersal kernel is fat-tailed due to an overrepresentation of long-distance dispersal events [36]. While also insect pollinated [37], the large flowers of the species and prolific nectar production suggest that *E. globulus* is adapted to bird pollination [38], which appears more common in the upper canopy [39]. The rare, strong-flying swift parrot (*Lathamus discolor*), which migrates annually from the mainland to the island of Tasmania to breed, is believed to be a key pollinator [40], [41] and disperser of pollen along the eastern distribution of *E. globulus* [42].

The geographic races of *E. globulus* on the east coast of Tasmania exhibit little differentiation in flowering time [43] which would favour gene flow, and indeed nuclear microsatellites reveal little neutral differentiation between the races in this region [44], [45], [46]. Nevertheless, the races in this continuous, core distribution of *E. globulus* on the eastern side of Tasmania are genetically differentiated in multiple functional [27] and fitness traits [28], arguing that selective gradients have generated significant adaptive differentiation. Such local adaptation suggests that long-distance pollen dispersal would result in mal-adapted hybrids. However, *E. globulus* exhibits severe inbreeding depression, which increases linearly with the relatedness between mates [47]. Thus, with local OP seed comprising selfs and varying levels of related matings, it has been argued that the deleterious effects of local inbreeding would favour the products of longer-distance pollen dispersal [33], [48].

In the present study, we address the hypothesis that the build-up of local inbreeding may counter local adaptation and favour the products of long-distance dispersal. We used a diallel mating scheme to produce intra- and inter-provenance crosses involving native *E. globulus* trees from southern-eastern and north-eastern regions of Tasmania, and monitored their survival and growth over a 13-year period in two field trials located within the native range of the species in south-eastern Tasmania.

## Materials and Methods

### Ethics statement

No permits or approvals were required for this field study, as the study involves a common tree species and was undertaken on private land which is not protected. Permission to access the trials was obtained from the land owners, which were Gunns Ltd. (Weilangta) and Forestry Tasmania (Geeveston). No endangered or protected species were sampled.

### Genetic material, crossing design, field trials and trait measurements

**Figure 1:**
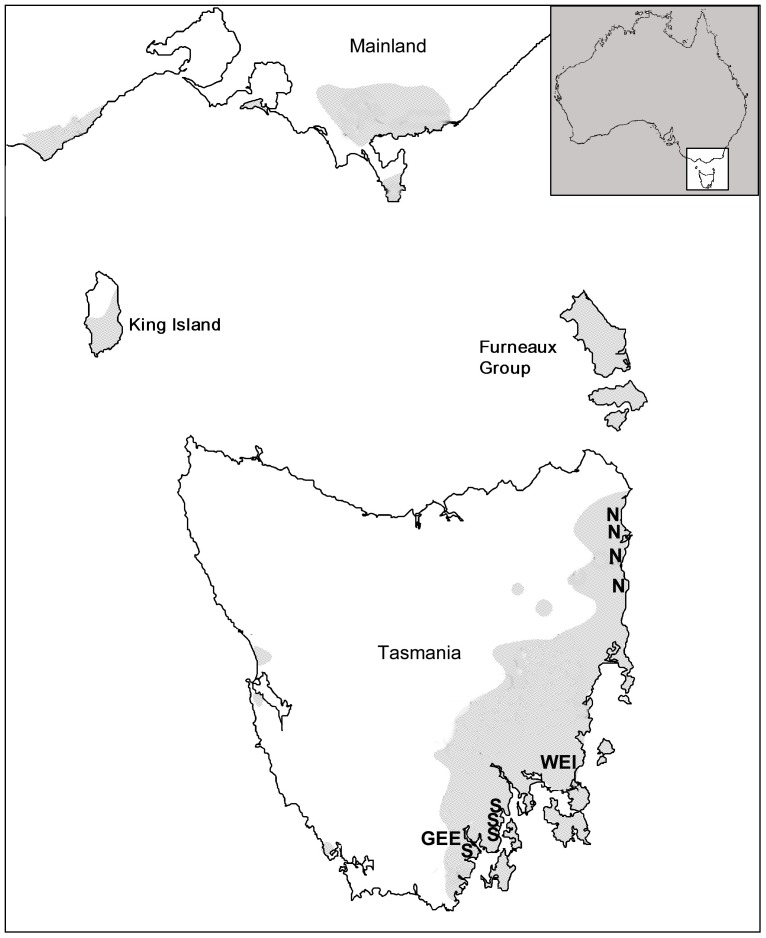
Location of native *E. globulus* trees and field trials in Tasmania. The wild parent trees that were used in the diallel and open-pollinated crosses are located in northern (N) and southern (S) Tasmania. The field trial locations in south-eastern Tasmania correspond to Geeveston (GEE) and Weilangta (WEI). The shaded area in the main map indicates the geographic range of *E. globulus*.

The genetic material, crossing design and field experimental layout have been described previously in the context of the assessment of nuclear and maternal effects on seed traits (seed mass and germination rate) and early growth performance in *E. globulus* [49]. Briefly, four trees were selected in each native stand in northern and southern Tasmania, and were crossed in a full 8×8 diallel mating scheme. The distance among the local northern trees averaged 26 km (minimum 9 km), among the local southern trees 16 km (minimum 4 km), and between northern and southern trees 216 km (minimum 183 km) (Figure 1). The diallel families thus represented four cross types corresponding to northern (♀_N_♂_N_) and southern (♀_S_♂_S_) regional crosses, and the long-distance, inter-regional crosses (i.e. inter-provenance hybrids) involving northern (♀_N_♂_S_) and southern (♀_S_♂_N_) females. Two other cross types were represented based on families derived from OP seed collected from the same wild trees used as parents in northern (♀_N_♂_OP_) and southern (♀_S_♂_OP_) Tasmania.

Progeny from the diallel cross and OP families were planted in two field trials, located in south-eastern (Weilangta; latitude 42° 44′ 44″, and longitude 147° 49′ 25″) and southern (Geeveston; latitude 43° 9′ 40″, and longitude 146° 51′ 38″) Tasmania (Figure 1). These trials sites fell within the geographic limits of the south-eastern and southern races of *E. globulus*, respectively (Figure 1), and consequently can be considered local sites for the southern (♀_S_♂_S_) regional crosses. The experimental layout of the field trials was a randomised block design with single-tree family plots, and comprising 17 and 18 replicates at Geeveston and Weilangta, respectively. For some families, the number of plants was insufficient to allow a complete distribution across the available number of replicates, and thus the missing positions within replicates were filled with individuals from other seedlots of the same cross type.

Breast-height diameter (DBH) growth and survival were assessed at ages 2, 4, 9 and 13 years from field planting. For survival, each tree was scored as either 1 (dead) or 0 (alive).

### Analysis of DBH growth

#### The general linear mixed model

Data analysis of DBH was undertaken by using the following general linear mixed model:
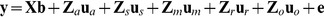
(1)where 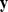 is a vector of individual tree observations, 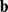 is a vector of fixed effects, 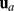 is a vector of random additive genetic effects, 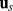 is a vector of random specific combining ability (SCA) effects, 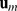 is a vector of random maternal effects, 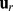 is a vector of random (non-maternal) reciprocal effects, 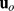 is a vector of additional random effects, 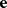 is a vector of random residuals, and 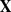, 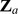, 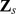, 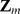, 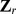 and 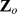 are incidence matrices relating the observations to the fixed and random effects. The joint distribution of the random terms was assumed to be multivariate normal, with mean vector zero and a variance matrix defined as a direct sum of the variance-covariance sub-matrices 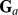, 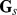, 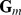, 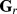, 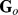 and **R** related to vectors 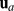, 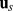, 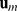, 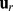, 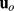 and 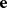, respectively.

#### Single-site analysis at a given age

A first single-site analysis of DBH aimed to compute and test at each age linear combinations of estimated means for the cross types involved in the diallel group of families. Fixed effects in 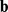 comprised a constant (mean) term, a factor with two levels to account for the effects of the diallel versus the OP groups of families, and a factor with four levels (i.e. the two regional ♀_N_♂_N_ and ♀_S_♂_S_ crosses, and the two inter-regional ♀_N_♂_S_ and ♀_S_♂_N_ hybrids) to account for the cross-type effects within the diallel group. The random effects in 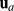, 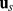, 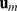 and 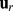 refer to model terms fitted within the diallel group, and the vector 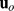 included sub-vectors for the effects of replicate and for the interaction between replicate and cross type.

The sub-matrix 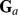 was specified as 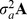, where 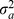 is the additive genetic variance and **A** denotes the matrix with additive genetic relationships among individuals. The effects within 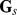, 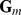 and 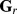 were assumed to be independent, and thus 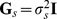, 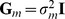 and 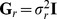, where 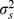, 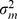 and 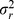 are variances associated with SCA, maternal and reciprocal effects, respectively, and 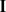 represents an identity matrix of the appropriate dimension. The random effects in the sub-vectors of 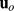 were assumed to be mutually independent, and thus 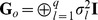, where 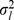 is the variance related to the *l*th effect in 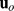 and 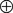 denotes the direct sum operation. The estimates obtained for these variance parameters will not be presented or discussed in detail, as the main interest of the data analysis is to estimate and test parameters involving the cross-type effects specified in 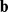. Nevertheless, modelling the random sources of variation may be important to compute unbiased estimates and accurate hypothesis tests of fixed model parameters (e.g. see [50]).

The vector of random residuals 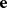 was partitioned into a sub-vector 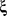, whose elements follow a spatially correlated process, and a sub-vector 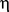 whose elements are spatially uncorrelated and distributed independently of 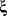. The general form of the variance-covariance structure for the effects in **e** was then defined as:
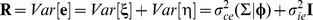
(2)where 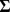 is the correlation matrix for the spatially dependent process (conditional on the parameters in vector 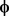) with the associated variance 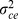, and 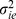 is the variance of the spatially uncorrelated residuals. The effects in 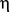 comprised two separate terms to account for variance heterogeneity between the diallel and OP groups. The effects in 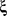 were modelled by using autoregressive structures in 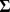. In this sense, and as proposed by Gilmour et al. [51], an initial step in the modelling procedure used a first-order autoregressive (AR1) structure that assumed separable spatially dependent processes in the row and column directions of the planting grid, and thus 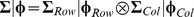, where 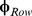 and 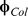 represent vectors for row and column autocorrelation parameters, respectively, and 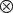 denotes the Kronecker product operation. For both trials, likelihood-ratio tests indicated that the separable AR1 structure provided always a significantly better fit than a model just assuming spatially uncorrelated effects in 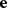. However, the separable AR1 structure may be less effective when competition, rather than environmental variation, becomes the dominant source of autocorrelation for effects in 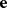 [52]. In this context, Stringer [52] and Stringer et al. [53] proposed to use an equal-roots third-order autoregressive (EAR3) structure in 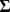 as a more efficient approach for jointly modelling both environmental variation and competition effects at the residual level. Therefore, the subsequent step in the modelling procedure was to apply a separable EAR3 structure in 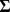 to evaluate whether this model would provide a significantly better fit than the separable AR1 structure. According to results from likelihood-ratio tests, this significant improvement was observed at ages 9 and 13 at Geeveston, and at age 13 at Weilangta, and thus the EAR3 structure was used in the final model for these cases. In order to simplify the modelling approach, we did not attempt to parameterize the dominant features of the spatial pattern to achieve stationarity for effects in 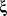, although non-stationary variation associated with global trend was identified in Weilangta (as judged by inspecting the sample variogram). Yet, extending the base separable AR1 structure to fit extraneous and global sources of variation has been shown to have a small impact on the estimation and ranking of breeding or clonal values [54], [55].

At a given trial and age, generalized least-squares means (LSM) were estimated for each of the four cross types within the diallel group. Then, linear combinations of LSM were constructed to estimate and test the following main effects expressed at the regional (provenance) level:
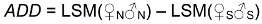
(3)




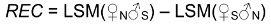
(4)




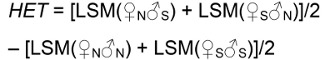
(5)where *ADD* =  net difference between the additive effects of the genes in the two regional populations, *REC* =  overall reciprocal effect (confounding maternal and non-maternal reciprocal effects at the regional level), and *HET* =  total mid-parent heterosis attributed to both inter-regional hybrids.

A second single-site analysis of DBH aimed to compute and test at age 13 years linear combinations involving the means of diallel and OP crosses of the same maternal origin, namely the two contrasts: ♀_N_♂_OP_ versus ♀_N_♂_N_, and ♀_S_♂_OP_ versus ♀_S_♂_S_. For this analysis, the fixed part of the model defined as in Equation (1) was re-parameterized so that vector 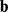 comprised a constant (mean) term and a factor with six levels, which pertain to all cross types irrespective of their group. The **G** and **R** matrices for random effects were specified as described above.

The (co)variance parameters were estimated by restricted maximum likelihood (REML), using the Average Information REML algorithm developed by Gilmour et al. [56]. Likelihood-ratio tests from preliminary analyses indicated that the REML variance estimate associated with the interaction between replicate and cross type was in general negligible or not significantly different from zero (*P*>0.05), and thus this random term was subsequently dropped in a final model. Hypothesis tests for linear combinations involving fixed effects in 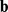 were carried out by using Wald *F*-statistics, with approximate denominator degrees of freedom being computed on the basis of the method proposed by Kenward and Roger [57].

#### Single-site analysis across ages

Using an extension of the mixed model defined as in Equation (1) for a given age, a repeated measures analysis was conducted for each site by combining DBH data across the four measurement ages in a multivariate form. The aim of this single-site analysis was to test the interaction of the effects *ADD*, *REC* or *HET* with age. To attain this goal, these effects were fitted in the fixed part of the mixed model by using linear contrasts based on the four levels of the cross-type factor within the diallel group. In this sense, the effects *ADD*, *REC* and *HET* were fitted as linear covariates in vector 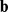, with the respective covariate values pertaining to the contrast coefficients of the LSM included in the combinations defined in Equations (3) to (5). Besides these effects, vector 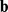 also comprised a term for the age means, a term to account for the diallel versus OP group effects at each age, and terms for the interactions of *ADD*, *REC* and *HET* with age.

For the random terms, the analysis was focused on modelling the effects in 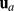 and 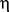, with their variance-covariance structures being specified as 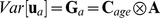 and 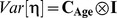, respectively, where 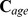 denotes an across-age (co)variance matrix. In general, the REML analysis could not converge when using an unstructured (co)variance matrix for 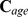, suggesting the need for a more parsimonious model. In this context, we have fitted and compared the following (co)variance matrices: uniform correlation model with heterogeneous variances, exponential correlation model with heterogeneous variances, first-order antedependence model and first-order factor analytical model (e.g. see [58], [59] for details on these models). A variance-covariance structure of the form 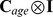 was also fitted for replicate effects in 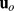, in which case we have simplified the analysis by assuming for 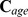 a uniform correlation model with heterogeneous variances. As the REML estimates of 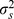, 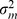 and 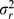 were in general small and not significant (*P*>0.05) under single-site analysis at a given age, a zero covariance across ages was assumed for effects in 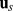, 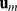 and 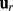. Thus, the variance-covariance structures 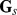, 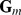 and 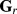 were all of the form 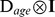, where 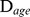 is a diagonal matrix with age variances as diagonal elements. The effects in 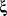 were assumed to be independent across ages (i.e. not involving an age covariance due to the trend process), and thus separate variance-covariance matrices of the form 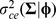 were fitted for different ages. To avoid convergence problems in the analysis across ages, we have restricted the (co)variance parameters for effects in 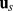, 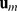, 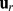 and 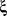 to remain fixed at the estimates previously obtained at a given age. Using the number of unrestricted (co)variance parameters that were estimated, the Akaike information criterion [60] was calculated to compare the different combinations of models fitted in 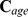 for effects in 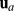 and 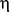: at Geeveston, the uniform correlation model with heterogeneous variances was selected for effects in both 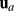 and 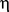; at Weilangta, the first-order factor analytical model and the exponential correlation model with heterogeneous variances provided the best fit for effects in 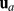 and 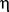, respectively.

Under the final across-age mixed model for each site, the interactions of *ADD*, *REC* or *HET* with age were tested by using conditional Wald *F*-statistics, with the computation of the denominator degrees of freedom being based on the approximation suggested by Kenward and Roger [57].

#### Across-site analysis at a given age

Analysis combining data across trials were conducted at each age to test whether the effects *ADD*, *REC* or *HET* interacted significantly with the trial site The mixed model applied here was an extension of that defined for single-site analysis. Vector 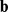 comprised a term for an overall mean, a term for a site effect, a term to account for the diallel versus OP group effects at each trial, the *ADD*, *REC* and *HET* effects and their interactions with site. The random effects in 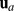 were fitted by using a variance-covariance structure specified as 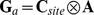, where 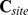 is an unstructured (co)variance matrix with additive genetic variances for each site as diagonal elements and the additive genetic covariance between sites on the off-diagonal. The random effects in 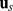, 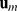, 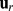 and 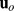 were assumed to be independent across sites, and the mixed model incorporated heterogeneity of variances between sites for each of these terms by using a variance-covariance structure of the form 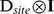, where 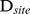 is a diagonal matrix with site variances as diagonal elements. The effects in 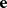 were also assumed to be independent across sites, and separate **R** variance-covariance structures were specified for individual sites as defined in Equation (2). The interactions between *ADD*, *REC* or *HET* and site were tested by using conditional Wald *F*-statistics, with the denominator degrees of freedom being calculated according to the approximation mentioned before.

### Analysis of survival

The analysis of survival used dead/alive data, and aimed to evaluate the differences in mortality rate between cross types. For binomial outcomes, when all of the cell counts (e.g. expected numbers of successes) exceed 5, the application of large-sample theory may result in reasonable estimates and reliable approximate inferences for model parameters based on maximum likelihood [61], [62]. Overall, our data set was sparse such that some cells (i.e. cross types) had small counts (i.e.≤5) and/or zeros for tree mortality across the examined ages, and thus the applicability of large-sample theory to our survival data may be uncertain. In particular, the cross ♀_N_♂_S_ was a source of quasi-complete separation in the Geeveston data, as the trees in this cross were all alive over time (i.e. 0 dead out of 77 trees in total). In this context, usual maximum likelihood estimates of parameter effects will be very large (or even infinite) and may be outweighed by their standard error estimates, leading to inflated *P*-values in hypothesis tests based on asymptotic theory [61]. Using data from the diallel group, we have indeed observed these results for the cross ♀_N_♂_S_ at Geeveston, by applying either: a) conventional maximum likelihood estimation under a logistic model including only the cross-type factor in the linear predictor; or b) Laplace approximation to the marginal likelihood under a generalized linear mixed model, which used a logit link function to relate the mean response to a linear predictor comprising cross type as a fixed effect, and replicate and full-sib family terms as random effects (i.e. a reduced model compared with that described in Equation (1), with the full-sib family term confounding the effects in 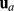, 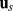, 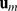 and 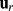).

To analyze the dead/alive data at each trial and age, we have explored the two options described below which are suitable when asymptotic results may not hold, such as with small cell counts and/or cases with less identifiable parameters due to complete or quasi-complete separation of the data. The following linear logistic model was fitted: 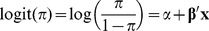
(6)where 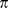 is the probability of an event of interest for a binary response variable 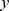 such that, for a binary outcome with value 1 pertaining to a dead tree, 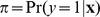; 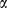 denotes an intercept term; and 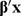 represents the linear predictor, with 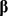 denoting the vector of effects to be estimated for the levels of the cross-type factor specified in 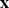. Under this model, we have considered the following two analytical alternatives:

- Penalized likelihood estimation, which leads to parameter estimates that are always finite and significantly less biased when compared with conventional maximum likelihood [63]. Penalized likelihood estimates of odd ratios (OR) and associated 95% profile-likelihood confidence limits were obtained for pairwise comparisons of cross types. Inference should be based on these confidence intervals when using penalized likelihood estimation, rather than relying on Wald-type confidence intervals or significance values from Wald chi-square statistics [64]. In this sense, for a OR involving a given pairwise comparison, a profile-likelihood confidence interval containing the value 1 indicates that the odds of death of the two cross types being compared are not significantly different (hence implying similar probabilities of survival).
- Exact inferential procedure, which has good small-sample properties providing valid inference results based on exact statistical tests [65]. Under this method, estimates of OR and related significance probabilities were obtained for pairwise comparisons of cross types. The calculation of these probabilities were based on likelihood ratio tests, using a (exact) conditional distribution generated for the sufficient statistics of the model parameters [66]. A correction was applied to make the likelihood ratio tests less conservative by compensating for the discreteness of a distribution, as suggested by Hirji et al. [67] for sparse data sets.

The linear predictor in the model defined in Equation (6) includes fixed effects only. Nevertheless, under the approach described above in (i), estimated variances of the Pearson residuals were close to one for both trials, indicating absence of overdispersion in the Bernoulli distribution assumed to describe the data. Besides indicating that the distribution specified for the data was adequate for both trials, the fact that the observations did not appear to be more dispersed than expected also suggests that the fitted model terms may be sufficient to explain the data.

ASReml [59] and SAS [68] were the software packages used in the data analyses.

## Results

**Figure 2:**
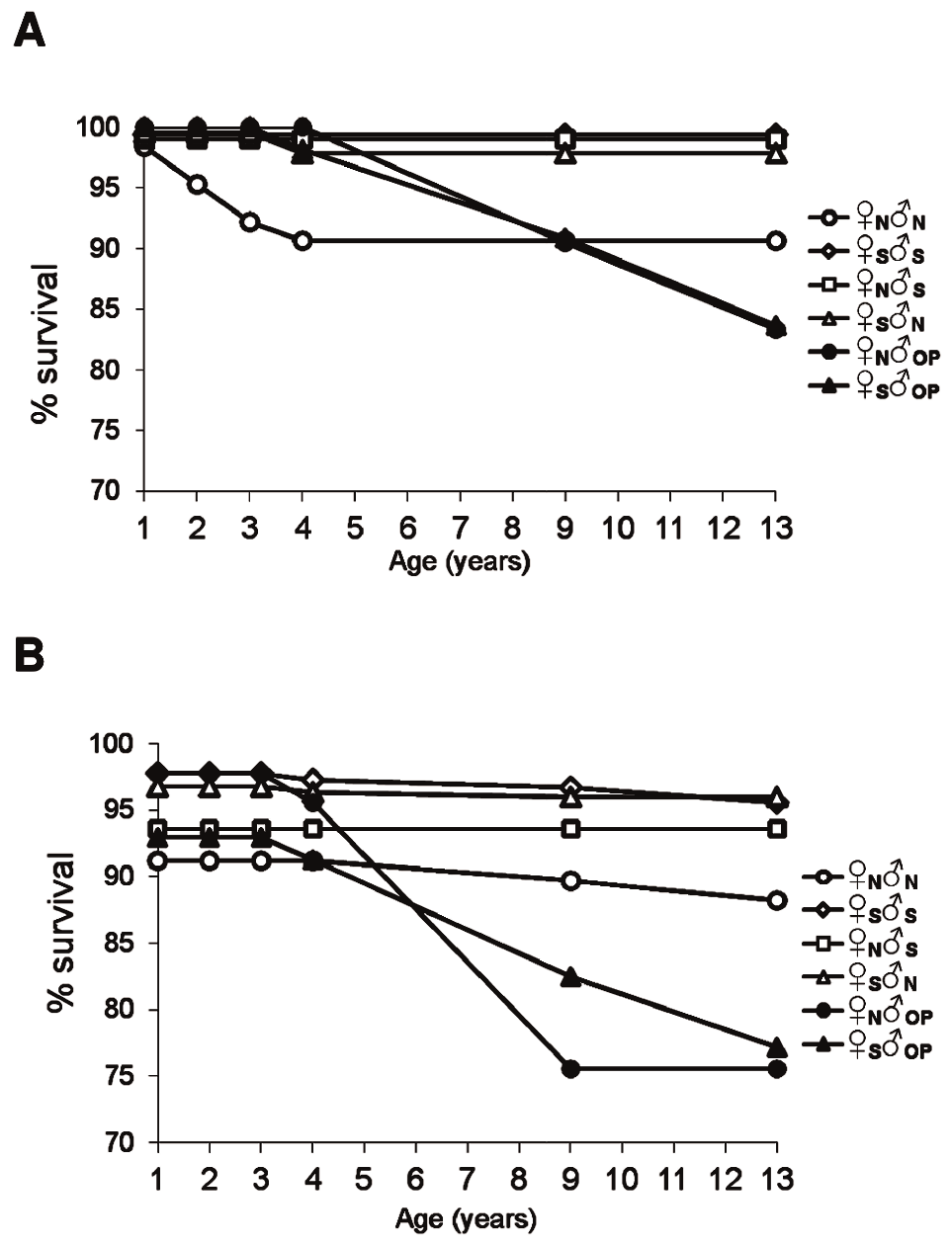
Age trend in population percentage survival for the *E. globulus* crosses. The crosses correspond to: northern (♀_N_♂_N_) and southern (♀_S_♂_S_) regional crosses, inter-regional hybrids involving northern (♀_N_♂_S_) and southern (♀_S_♂_N_) females, and open-pollinated (OP) families collected from the same wild parent trees used as parents in northern (♀_N_♂_OP_) and southern (♀_S_♂_OP_) Tasmania. The progenies from these crosses were tested at the Geeveston (A) and Weilangta (B) trial sites.

**Figure 3:**
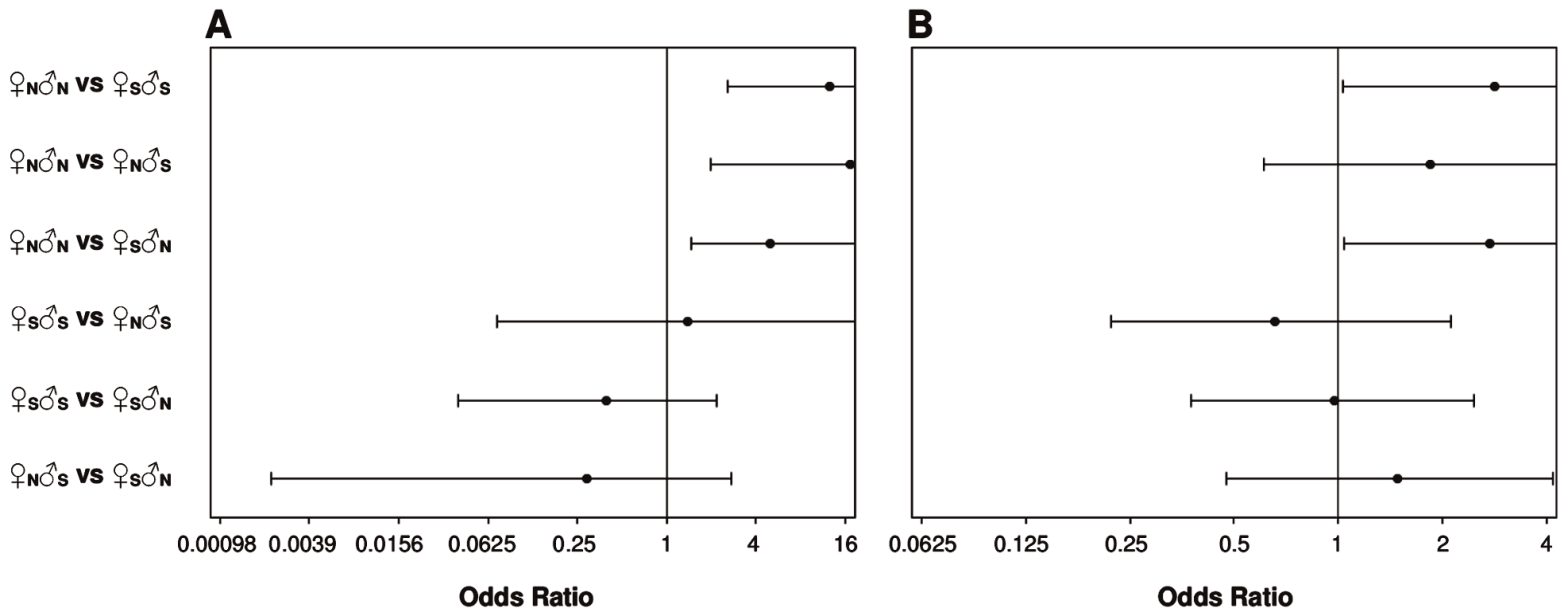
Comparisons of differences in mortality for pairs of *E. globulus* crosses within the diallel group of families. The comparisons pertain to age 13 years from field planting at the Geeveston (A) and Weilangta (B) trial sites, and are based on penalized likelihood estimates of odds ratios (OR) under a logistic model. OR estimates (represented by the symbol •) with 95% profile-likelihood confidence limits are given for each pair of crosses, which correspond to: northern (♀_N_♂_N_) and southern (♀_S_♂_S_) regional crosses, and inter-regional hybrids involving northern (♀_N_♂_S_) and southern (♀_S_♂_N_) females. The OR axis displays a base 2 logarithmic scale. A confidence interval for the OR containing the value 1 (marked with a vertical line) indicates that the odds of death of the two crosses being compared are not significantly different, implying similar probabilities of survival. In this sense, as the upper confidence limit is not of particular interest, the range of the confidence limits is truncated for a more clear visualization.

The ♀_N_♂_N_ non-local regional crosses were less well adapted to the planting sites than the ♀_S_♂_S_ local regional crosses, as illustrated by the age trend in population percentage survival (Figure 2). Figure 3 shows the penalized likelihood estimates of OR and associated 95% profile-likelihood confidence limits for pairwise comparisons involving the cross types within the diallel group at age 13 years (for pairwise comparisons at ages 2, 4 and 9 years, see Figure S1), and indicates that the odds of death of the ♀_N_♂_N_ crosses tended to be significantly greater than the odds of death of the ♀_S_♂_S_ crosses. In this sense, the differences between the two regional cross types in the probabilities of survival were in general larger at the most southerly site at Geeveston than at Weilangta. Inference results for pairwise comparisons provided similar conclusions when OR were based on either penalized likelihood estimation (Figures 3 and S1) or exact statistical tests (Table S1). There was also a trend for the DBH of ♀_N_♂_N_ survivors to be lower than surviving ♀_S_♂_S_ trees at both sites (i.e. the *ADD* effects; Table 1). Nevertheless, the magnitudes of these differences for DBH were never found to be statistically significant (*P*>0.05) within sites at a given age (Table 1), neither led to significant interactions with age within sites (Table 2) or with site at a given age (Table 3).

**Table 1:** Estimates of effects (with significance probabilities given in parenthesis) expressed at the regional (provenance) level, based on linear combinations of least-squares means that were calculated for *E. globulus* intra- and inter-regional crosses.

	Geeveston				Weilangta			
	2 yr	4 yr	9 yr	13 yr	2 yr	4 yr	9 yr	13 yr
*ADD*	0.22 (*P*>0.10)	−0.02 (*P*>0.10)	−1.04 (*P*>0.10)	−1.22 (*P*>0.10)	0.41 (*P*>0.10)	−0.29 (*P*>0.10)	−0.98 (*P*>0.10)	−1.06 (*P*>0.10)
*REC*	0.16 (*P*>0.10)	0.05 (*P*>0.10)	−0.17 (*P*>0.10)	−0.17 (*P*>0.10)	0.29 (*P*>0.10)	0.56 (*P* = 0.05)	0.80 (*P* = 0.05)	1.17 (*P* = 0.04)
*HET*	0.06 (*P*>0.10)	0.11 (*P*>0.10)	0.53 (*P* = 0.10)	0.89 (*P* = 0.04)	0.18 (*P*>0.10)	0.52 (*P* = 0.009)	1.44 (*P*<0.001)	2.27 (*P*<0.001)

The results (in cm) are based on single-site analysis undertaken for breast-height diameter (DBH) at a given age from field planting (i.e. 2, 4, 9 or 13 years) for each of the trial sites (Geeveston and Weilangta).

*ADD* =  net difference between the additive effects of the genes in the intra-regional ♀_N_♂_N_ and ♀_S_♂_S_ crosses.

*REC* =  overall reciprocal effect (confounding maternal and non-maternal reciprocal effects at the regional level).

*HET* =  total mid-parent heterosis attributed to both inter-regional ♀_N_♂_S_ and ♀_S_♂_N_ hybrids.

**Table 2:** Results from significance tests (Wald *F*-statistics, with associated significance probabilities given in parenthesis) for the interactions of the regional-level *ADD*, *REC* or *HET* effects with age.

	Geeveston	Weilangta
*ADD* × age	0.25 (*P*>0.10)	0.93 (*P*>0.10)
*REC* × age	0.29 (*P*>0.10)	2.43 (*P* = 0.06)
*HET* × age	1.33 (*P*>0.10)	10.54 (*P*<0.001)

The results are based on a repeated measures analysis that was conducted for each trial site (Geeveston or Weilangta) by combining breast-height diameter (DBH) data across four measurement ages (i.e. 2, 4, 9 and 13 yearsfrom field planting).

*ADD* =  net difference between the additive effects of the genes in the intra-regional ♀_N_♂_N_ and ♀_S_♂_S_ crosses.

*REC* =  overall reciprocal effect (confounding maternal and non-maternal reciprocal effects at the regional level).

*HET* =  total mid-parent heterosis attributed to both inter-regional ♀_N_♂_S_ and♀_S_♂_N_ hybrids.

**Table 3:** Results from significance tests (Wald *F*-statistics, with associated significance probabilities given in parenthesis) for the interaction of the regional-level *ADD*, *REC* or *HET* effects with trial site.

	2 yr	4 yr	9 yr	13 yr
*ADD* × site	0.22 (*P*>0.10)	0.37 (*P*>0.10)	0.01 (*P*>0.10)	0.01 (*P*>0.10)
*REC* × site	0.10 (*P*>0.10)	2.17 (*P*>0.10)	2.79 (*P* = 0.10)	3.16 (*P* = 0.08)
*HET* × site	0.53 (*P*>0.10)	2.98 (*P* = 0.09)	4.58 (*P* = 0.04)	5.45 (*P* = 0.02)

For each age (i.e. 2, 4, 9 or 13years from planting), the results are based on an across-site analysis that was conducted by combining breast-height diameter (DBH) data measured at the Geeveston and Weilangta trial sites.

*DD* =  net difference between the additive effects of the genes in the intra-regional ♀_N_♂_N_ and ♀_S_♂_S_ crosses.

*REC* =  overall reciprocal effect (confounding maternal and non-maternal reciprocal effects at theregional level).

*HET* =  total mid-parent heterosis attributed to both inter-regional ♀_N_♂_S_ and ♀_S_♂_N_ hybrids.

The significant differences found in mortality between the ♀_N_♂_N_ and ♀_S_♂_S_ crosses (Figures 3 and S1; Table S1), however, were not evident in their OP progenies where, by age 13-years, there was little difference in mortality (Figure 2). Yet, there was clear evidence that the performance of these OP progenies was dominated by inbreeding depression. There were evident losses in the survival of the OP progeny relative to their regional crosses (i.e. ♀_N_♂_OP_ versus ♀_N_♂_N_, and ♀_S_♂_OP_ versus ♀_S_♂_S_) at age 13 years (Figure 2), with exact statistical tests of OR being significant (*P*≤0.05) in these comparisons except for ♀_N_♂_OP_ versus ♀_N_♂_N_ (*P*>0.05) at Geeveston. This latter result is in agreement with a less adapted ♀_N_♂_N_ non-local regional cross at the most southerly site at Geeveston, as also suggested by the inference results for survival based on the differences between the two regional cross types at this site (Figures 3 and S1; Table S1). For DBH, the growth of the OP progeny was gradually reduced with increasing age relative to their regional crosses at both sites, with the contrasts ♀_N_♂_OP_ versus ♀_N_♂_N_ and ♀_S_♂_OP_ versus ♀_S_♂_S_ revealing DBH reductions ranging from 16% to 23% (significant at *P*≤0.05) at age 13 years; as for survival, there was also little difference at both sites in the DBH growth of the OP progenies at age 13 years (data not shown).

The superior performance of outcrosses was even more obvious when they arose from long-distance pollination. There was certainly no evidence that the long-distance ♀_N_♂_S_ and ♀_S_♂_N_ crosses experienced outbreeding depression, despite the previous evidence for adaptive differences between the non-local ♀_N_♂_N_ and local ♀_S_♂_S_ regional crosses. Rather, the long-distance crosses exhibited equivalent (♀_S_♂_N_ versus ♀_S_♂_S_ at both sites; ♀_N_♂_S_ versus ♀_N_♂_N_ at Weilangta) or significantly lower (♀_N_♂_S_ versus ♀_N_♂_N_ at Geeveston) mortality when compared with the regional crosses (Figures 3 and S1; Table S1), and showed significant (*P*≤0.05) positive mid-parent heterosis (i.e. the *HET* effects) for DBH at both sites (Table 1). However, the development over time of mid-parent heterosis for DBH (Table 1) was site dependent (Table 2). At Geeveston, the mean DBH of the long-distance crosses was only marginally (at the 5% significance level) greater than the mid-parent value by age 13 years, but at Weilangta highly significant (*P*≤0.001) heterosis was evident by age 9 years (Table 1). Furthermore, at age 13 years, the ♀_N_♂_N_ cross had worse DBH growth than either of the long-distance crosses at both sites, with the pairwise comparisons being in general significant (*P*≤0.05); although a similar tendency was also observed for the ♀_S_♂_S_ cross, its differences with the long-distance crosses were smaller in magnitude, and only the pairwise comparison involving the contrast ♀_S_♂_S_ versus ♀_N_♂_S_ at Weilangta was found to be significant at the 5% level (data not shown). Interactions of the *HET* effects with site for DBH were also found to be significant (*P*≤0.05) at ages 9 and 13 years (Table 3).

Marginal statistically significant differences at the 5% level were observed between the DBH of the reciprocal long-distance crosses (♀_N_♂_S_ versus ♀_S_♂_N_) at Weilangta only (Table 1). In this sense, the *REC* effects were significant after age 2 years, due to the long-distance crosses with northern females (♀_N_♂_S_) outperforming those with southern females (♀_S_♂_N_). These differences between sites in the reciprocal long-distance crosses led to marginal effects (i.e. statistically significant at the *P*≤0.10 only) for the interactions of *REC* with age (Table 2) and with site (Table 3).

## Discussion

The 13-year survival and (to a less extent) growth data indicated selection against the non-local regional cross (i.e. ♀_N_♂_N_ versus ♀_S_♂_S_) at both of the examined southern trial sites, consistent with local adaptation [69]. While specific functional trait differences were not assessed in this study, broad-scale, latitudinal clines in numerous functional traits occur within the continuous, core distribution of *E. globulus* in eastern Tasmania [27]. Common-environment field trials based on OP progenies collected from native populations indicated that there is marked quantitative genetic differentiation between northern and southern races, with northern populations having greater bark thickness [27]; wood with more extractives and lignin content, and a higher syringyl/guaiacyl ratio [70]; later transition to adult foliage [71]; more resistance to leaf disease [72] and drought [28]; and tending to be generally slower growing than southern races [73], [74]. There are also differences in foliar chemistry [42], [75], [76] and marsupial herbivory [77] that could partly explain the fitness differences we have observed. A specific study undertaken on the Geeveston trial at age 3 years showed that the northern trees (♀_N_♂_N_) were more susceptible to marsupial herbivory than southern trees (♀_S_♂_S_) in the field and in artificial feeding trials, most likely due to their foliage containing more tannins and essential oils [78]. However, this does not explain the patterns of mortality in the slower growing OP progenies, where mortality occurred later, continued to increase substantially with age, and was eventually greater than the regional crosses, but not significantly different between regions (i.e. ♀_N_♂_OP_ versus ♀_S_♂_OP_) at age 13 years.

The higher mortality and reduced growth in the OP progenies is consistent with the onset of the deleterious effects of inbreeding depression, now shown to occur in five independent populations of wild OP progenies of *E. globulus* [33], [34], [35], [79], [80], [81]. This is consistent with molecular studies that showed mixed mating and bi-parental inbreeding [36]. Therefore, a large-component of the reduced fitness of the OP progenies is no doubt reflecting selection against the products of self-fertilization. Mixed mating systems are common in flowering plants [82]. While providing reproductive assurance, strong post-dispersal selection against progenies derived from self pollination in many forest tree species [48], [83], including *E. globulus* [33], [34], [35], [47], [79], [81], would argue that, when competing with outcrosses, selfs normally contribute little to subsequent generations [35].

Selection against related near-neighbour pollinations is also expected to be included in the reduced fitness of our OP progenies. This would be expected as: (i) there is local spatial clustering of related individuals in a *E. globulus* forest, which extends over 20 to 45 m (i.e. one to two tree heights), and is believed to result primarily from limited seed dispersal [31], [32]; (ii) there is an exponential decay in the probability of pollination [36], arguing for a predominance of near-neighbour (within approximately 50 m) pollinations; (iii) significant bi-parental inbreeding has been detected in seedlings from OP progenies (0.04 to 0.11; [36]); and (iv) inbreeding depression for growth and survival has been shown to linearly increase with relatedness [47]. Indeed, significant inbreeding depression has been reported in crosses with nearest flowering neighbours (average distance apart 25 m), compared with crosses involving *E. globulus* trees separated by just 250 m [33]. In addition to the inbreeding in OP progenies resulting in selection favouring the products of local outcrossing, the present study shows that significant mid-parent heterosis for growth also occurs in the F_1_ hybrids derived from the long-distance crosses (over an average distance of 216 km in the present case). However, for the two southern trial sites studied, offspring derived from the long-distance ♀_S_♂_N_ hybrid cross did not differ significantly in their survival and growth performances to the local (i.e. with the same southern maternal origin) regional ♀_S_♂_S_ cross, despite mal-adaptation of the non-local regional ♀_N_♂_N_ population.

Post-dispersal selection against the products of biparental inbreeding will be expected to increase the realised pollen-mediated gene flow, by favouring progeny derived from long-distance crosses and/or from local pollinations involving distal trees [84]. Such selection against the products of selfing and bi-parental inbreeding have been shown to result in a 60% increase in effective pollen dispersal distances between the seedling and sapling stages of the tree *Aesculus turbinate* [85]. The changing nature of gene flow across different life stages has also been emphasised in a recent study of the tropical tree *Prunus africana* [86]: a significant increase in effective paternal gene dispersal distances was detected in a natural population from seeds, and from established young seedlings to middle-aged seedlings; increases were also detected in effective maternal gene dispersal distances across successive recruitment stages. Thus, as with pollen, seed dispersal itself may also underestimate seed-mediated gene flow due to density dependent selection or predation pressure favouring the products of longer distance seed dispersal [86], [87], [88].

The selection against the products of inbreeding varies with population and site, and accumulates with age in *E. globulus* [47], which also implies variability in the discrepancy between the pre- and post-selection estimates of pollen-mediated gene flow. Similarly, the magnitude of heterosis for DBH in the studied long-distance crosses was also age (Table 2) and site (Table 3) dependent. Environment-dependent heterosis is well recognised in both animals [89], [90] and plants [91], including forest trees [92]. At the inter-specific level, heterosis in hybrid *Eucalyptus* is thought to often reflect trait complementarily and be most manifest when hybrids are grown in zones which are marginal for the pure species [93], [94]. This effect could be operating at the provenance-level in the present case, and could also explain the greater mid-parent heterosis observed for DBH in the F_1_ hybrids at our more northern trial at Weilangta, which deviates geographically from both of the regional populations sampled. However, while the mean DBH of either of the F_1_ hybrid crosses was greater than the local regional southern cross at this site, by age 13 years, the difference between the ♀_S_♂_S_ and ♀_S_♂_N_ crosses was not significant at the 5% level (i.e. the ♀_S_♂_S_ regional cross did only differ significantly from the ♀_N_♂_S_ hybrid cross, which involves the northern females).

The fitness of inter-population hybrids determines the potential for long-distance pollen dispersal to redistribute genetic variation within a species in response to climate change [5], as well as to recover genetic diversity following population bottlenecks (e.g. founder events - [95], [96], [97]. It also affects long-term fitness of populations established following anthropogenic assisted gene flow amongst populations for climate change mitigation and restoration purposes [98], [99], [100]. Nevertheless, there is a paucity of long-term studies of the genetic consequences of inter-population crossing [101], particularly in forest trees [5], [100]. Most studies with forest trees have involved northern hemisphere gymnosperms, and few studies extended beyond the first generation. These studies, reviewed in Harfouche et al. [102], showed variable levels of heterosis but, in most cases, the inter-provenance F_1_ hybrids were intermediate in their performance. There are a few cases of outbreeding depression reported in the first generation of inter-provenance hybrids in forest trees, and these were most due to crossing highly ecologically divergent provenances (i.e. extrinsic outbreeding depression [100]). For example, a recent study of *Abies sachalinensis* in Japan showed selection against a non-local, high altitude provenance when grown on a low altitude site, and outbreeding depression (below mid-parent performance) in the F_1_ hybrids between high and low altitude populations [103]. In the case of *Pinus pinaster*, where 10 provenances were crossed in a diallel manner, significant mid-parent heterosis was observed for tree height, and there was a significant positive correlation between genetic divergence between provenances and heterosis [102]. In the case of *E. globulus*, several crossing programs [104], [105] provided strong evidence of significant mid-parent heterosis following crossing of widely separated provenances. Indeed, the present study argues that heterosis may be sufficient to counter mal-adaptation of non-local provenances (as indicated by the poorer survival and DBH performances of the ♀_N_♂_N_ cross relative to the long-distance crosses at both sites), and prevent selection against the products of long-distance dispersal in the first generation.

The heterosis we observed in *E. globulus* following the long-distance crossing could not be due to a release from a high level of inbreeding, as the distance between the parents used in regional crosses far exceeds the family group structure in native *E. globulus* forests [31]. The study of proximity-dependent crossing in *E. globulus* by Hardner et al. [33] suggested that, once parents were separated by 250 m (beyond the family group), there was no detectable inbreeding depression in their progeny, and little proximity-dependent change in performance thereafter (i.e. from 250 m to 100 km). However, subtle differences in performance could not be dismissed entirely, as only OP progeny were available as controls and it was not possible to accurately separate the male locality effect from heterosis (as also in the present study). Thus, there is the possibility that the observed heterosis may be due to a low-level of inbreeding associated with broader-scale gene flow within regions. There is a gradual decline in genetic similarity with distance in *E. globulus* after the family group structure is transgressed [31], [45], consistent with an isolation-by-distance model [46]. Indeed, Yeoh et al. [45] suggested that pollen-mediated gene flow may extend the population to beyond 40 km (within the local southern and northern regions, our parent trees were on average 16 and 26 km further apart, respectively).

Another possible cause of the observed heterosis may lie in the specific inheritance patterns of traits associated with the mal-adaptation of the northern population. For example, many of the foliar chemical mean differences between the northern and southern trees studied were inherited in a dominant manner in the F_1_ hybrids [78]. Variation in functional traits where means are inherited in a dominant manner could thus lead to heterosis in fitness traits. Alternatively, if the susceptibility to marsupial browsing is a contributor to the mal-adaptation of the non-local regional ♀_N_♂_N_ cross at the examined southern sites, it is possible that the browsing levels in the long-distance F_1_ hybrids and local regional ♀_S_♂_S_ cross are insufficient to affect tree growth. In a previous study, the ♀_N_♂_S_ and ♀_S_♂_N_ hybrids at the Geeveston trial exhibited intermediate levels of marsupial browsing on a logarithmic scale, meaning that they were more similar to the resistant local ♀_S_♂_S_ population on the untransformed scale [78], which may be reflected in the fitness response. Bias towards the ♀_S_♂_S_ cross could be further accentuated if threshold levels of defoliation are required before the growth of *E. globulus* is affected. Such defoliation thresholds have been reported in *E. globulus*, and can vary with environment and type of defoliation [106]. Thus, the observed heterosis could arise if neither the F_1_ hybrids nor the local ♀_S_♂_S_ cross exceeded a defoliation threshold but the non-local ♀_N_♂_N_ cross did. It is also possible that site differences in the observed mid-parent heterosis may reflect different defoliation thresholds of the ♀_N_♂_N_ cross at the two trials.

There was some evidence of a reciprocal effect on DBH after age 2 years at the Weilangta site, but this was not significant at any age at the Geeveston site (Table 1). While maternal effects were evident in nursery traits in these crosses, this was mainly associated with seed size and germination differences, and rapidly decreased following field planting [49]. The crosses were undertaken in the natural stands, and it is possible that reciprocal effects could arise through epigenetic effects transmitted from the parents or seed development. However, epigenetic effects are quite dynamic through plant development [107], and the phenotypic effects of trans-generational or “memory” epigenetic effects would need to be transmitted only through the male or female parent and increase with age, in order to be consistent with the reciprocal effects on growth that we have observed at Weilangta. The chloroplast genotypes of the parents used in our crosses differ, with the northern parents having either the J_c_ or J_et_ chloroplast types and the southern parents having the J_s_ type [49], consistent with the geographic distribution of these chloroplast types in the native *E. globulus* population in eastern Tasmania [108]. As is generally the case in angiosperms, including eucalypts, the chloroplasts are maternally inherited in these crosses [109]. While detected through the difference between the ♀_N_♂_S_ and ♀_S_♂_N_ crosses, the overall reciprocal effect we have observed was due mainly to the strong interactive effect of a single parent (i.e. the only one with the J_et_ haplotype; data not shown) and was site-specific. Such site- or environment-specific expression of reciprocal effects has been previously reported for inter-specific F_1_ hybrids [110], [111], [112], [113], and could be due to differences in the chloroplast genome and/or a cytonuclear interactions which may become more evident with age.

The fitness of inter-provenance hybrids in subsequent generations is one of the issues affecting decisions on the use and movement of non-local provenances of forest trees due to the potential for outbreeding depression [100]. All else being equal, subsequent generations would be expected to decline in fitness due to a decay in any dominance variation underlying heterosis, as well as due to the appearance of deleterious epistatic interactions if coadapted gene complexes had been disrupted [22]. There is ample evidence that epistasis contributes to population differentiation in plants [114], [115], [116], [117], but little empirical evidence for forest trees [118], [119]. Epistatic differentiation may accumulate through randomly changing the genetic background, such as when periods of isolation (i.e. founder events) and genetic drift occur [120]. However, Kremer et al. [5] suggested that, as tree populations are seldom isolated from each other, outbreeding depression arising from adverse gene interactions is likely to be rare. Epistasis may also accompany differentiation under strong selection [121], and multi-locus simulations have shown that population differentiation in quantitative traits is first driven by changes in the covariance amongst alleles from different loci and subsequently followed by changing allelic frequencies [122]. While outbreeding depression arising through the breakup of favourable epistatic interactions within gene complexes appears to occur in inter-specific hybrids with *E. globulus* [119], significant epistatic variance for growth has not been in general detected within a breeding population from a single race of *E. globulus* [123], [124]. Nevertheless, there is the possibility that epistasis could be revealed with greater power, as for example in targeted line-cross analyses of inter-provenance differentiation [121], which are noticeably rare for forest trees.

## Supporting Information

Figure S1 **Comparisons of differences in mortality for pairs of** ***E. globulus*** **crosses within the diallel group of families.** The comparisons pertain to ages 2, 4 and 9 years from field planting at the Geeveston and Weilangta trial sites, and are based on penalized likelihood estimates of odds ratios (OR) under a logistic model. OR estimates (represented by the symbol •) with 95% profile-likelihood confidence limits are given for each pair of crosses, which correspond to: northern (♀_N_♂_N_) and southern (♀_S_♂_S_) regional crosses, and inter-regional hybrids involving northern (♀_N_♂_S_) and southern (♀_S_♂_N_) females. The OR axis displays a base 2 logarithmic scale. A confidence interval for the OR containing the value 1 (marked with a vertical line) indicates that the odds of death of the two crosses being compared are not significantly different, implying similar probabilities of survival. In this sense, as the upper confidence limit is not of particular interest, the range of the confidence limits is truncated for a more clear visualization.
(TIF)

Table S1 **Comparisons of differences in mortality for pairs of** ***E. globulus*** **crosses within the diallel group of families.** Odd ratios (OR) were estimated under a logistic model using exact inference on the parameters, and the significance probabilities of the OR are given for each pair of crosses being compared, which correspond to: northern (♀_N_♂_N_) and southern (♀_S_♂_S_) regional crosses, and inter-regional hybrids involving northern (♀_N_♂_S_) and southern (♀_S_♂_N_) females. The probabilities are based on likelihood ratio tests using a (exact) conditional distribution generated for the sufficient statistics of the model parameters. A correction was applied to make the likelihood ratio tests less conservative by compensating for the discreteness of a distribution, as suggested by Hirji et al. (see [67] in the References section) for sparse data sets.
(PDF)
