# Supplementary material for: Heterosis May Result in Selection Favouring the Products of Long-Distance Pollen Dispersal in Eucalyptus
Source: PLoS One. 2014 Apr 21;9(4):e93811. doi: 10.1371/journal.pone.0093811 (PMC3994164; doi:10.1371/journal.pone.0093811)
Supplement: Table S1 — Comparisons of differences in mortality for pairs of E. globulus crosses within the diallel group of families. Odd ratios (OR) were estimated under a logistic model using exact inference on the parameters, and the significance probabilities of the OR are given for each pair of crosses being compared, which correspond to: northern (♀N♂N) and southern (♀S♂S) regional crosses, and inter-regional hybrids involving northern (♀N♂S) and southern (♀S♂N) females. The probabilities are based on likelihood ratio tests using a (exact) conditional distribution generated for the sufficient statistics of the model parameters. A correction was applied to make the likelihood ratio tests less conservative by compensating for the discreteness of a distribution, as suggested by Hirji et al. (see [67] in the References section) for sparse data sets. (PDF) [file pone.0093811.s002.pdf]

Table S1. Comparisons of differences in mortality for pairs of *E. globulus* crosses within the diallel group of families. The comparisons pertain to ages 2, 4, 9 and 13 years from field planting at the Geeveston and Weilangta trial sites. Odd ratios (OR) were estimated under a logistic model using exact inference on the parameters, and the significance probabilities of the OR are given for each pair of crosses being compared, which correspond to: northern (♀$_N$♂$_N$) and southern (♀$_S$♂$_S$) regional crosses, and inter-regional hybrids involving northern (♀$_N$♂$_S$) and southern (♀$_S$♂$_N$) females. The probabilities are based on likelihood ratio tests using a (exact) conditional distribution generated for the sufficient statistics of the model parameters. A correction was applied to make the likelihood ratio tests less conservative by compensating for the discreteness of a distribution, as suggested by Hirji et al. (see [67] in the References section) for sparse data sets.

Age (years)	Comparison	Geeveston	Weilangta
2	♀$_N$♂$_N$ – ♀$_S$♂$_S$	(0.069)	(0.035)
	♀$_N$♂$_N$ – ♀$_N$♂$_S$	(0.091)	(NS)
	♀$_N$♂$_N$ – ♀$_S$♂$_N$	(0.046)	(NS)
	♀$_S$♂$_S$ – ♀$_N$♂$_S$	(NS)	(NS)
	♀$_S$♂$_S$ – ♀$_S$♂$_N$	(NS)	(NS)
	♀$_N$♂$_S$ – ♀$_S$♂$_N$	(NS)	(NS)
4	♀$_N$♂$_N$ – ♀$_S$♂$_S$	(0.007)	(0.062)
	♀$_N$♂$_N$ – ♀$_N$♂$_S$	(0.018)	(NS)
	♀$_N$♂$_N$ – ♀$_S$♂$_N$	(0.004)	(NS)
	♀$_S$♂$_S$ – ♀$_N$♂$_S$	(NS)	(NS)
	♀$_S$♂$_S$ – ♀$_S$♂$_N$	(NS)	(NS)
	♀$_N$♂$_S$ – ♀$_S$♂$_N$	(NS)	(NS)
9	♀$_N$♂$_N$ – ♀$_S$♂$_S$	(0.002)	(0.046)
	♀$_N$♂$_N$ – ♀$_N$♂$_S$	(0.008)	(NS)
	♀$_N$♂$_N$ – ♀$_S$♂$_N$	(0.008)	(NS)
	♀$_S$♂$_S$ – ♀$_N$♂$_S$	(NS)	(NS)
	♀$_S$♂$_S$ – ♀$_S$♂$_N$	(NS)	(NS)
	♀$_N$♂$_S$ – ♀$_S$♂$_N$	(NS)	(NS)
13	♀$_N$♂$_N$ – ♀$_S$♂$_S$	(0.002)	(0.054)
	♀$_N$♂$_N$ – ♀$_N$♂$_S$	(0.008)	(NS)
	♀$_N$♂$_N$ – ♀$_S$♂$_N$	(0.016)	(0.053)
	♀$_S$♂$_S$ – ♀$_N$♂$_S$	(NS)	(NS)
	♀$_S$♂$_S$ – ♀$_S$♂$_N$	(NS)	(NS)
	♀$_N$♂$_S$ – ♀$_S$♂$_N$	(NS)	(NS)

NS = $P > 0.10$
